# Supplementary material for: Identification of Oocyst-Driven Toxoplasma gondii Infections in Humans and Animals through Stage-Specific Serology—Current Status and Future Perspectives
Source: Microorganisms. 2021 Nov 13;9(11):2346. doi: 10.3390/microorganisms9112346 (PMC8618849; doi:10.3390/microorganisms9112346)
Supplement: Supplementary file 1 [file microorganisms-09-02346-s001.zip › microorganisms-1429330-supplementary/Table S1.pdf]

Table S1. IgM and IgG avidity tests employed in livestock*

	Assay format (Antigen employed)	Ref. test	Pig sera								Results	Ref.
			(N/E)	Host-dependent factors				Parasite-dependent factors				
				Nº	Age	Pig**	Samplings	*T. gondii* strain	Stage	Dose		
**IgM tests**	Indirect and reverse ELISA (whole TZ extract)	TZ and SAG1 ELISAs	E	6	8 w	SPF	0-133 dpi	RH	TZ	10^6	All groups:	[1]
			E	6	8 w	SPF	0-133 dpi	SSI119	TZ	na	- IgG seroconversion: 1–2 wpi	
			E	9	na	na	0-98 dpi	SSI 119	Oo	10^3	- IgG levels stabilized: 3–6 wpi	
			E	10	na	na	0-98 dpi	SSI 119	Oo	10^4	- IgG persisted throughout the study period	
			E	10	na	SPF	0-98 dpi	SSI 119	TC	na	- IgM appeared at 7dpi	
			E	10	na	SPF	0-98 dpi	R92	TC	na	- IgM peaked at 10 dpi and lived up to 21-24 dpi	
**Avidity tests**	ELISA (whole TZ extract)	TZ ELISA	E	1	Piglet	Duroc	0-140 dpi	RH	TZ	10^7	High avidity values: 75–100 dpi	[2]
	ELISA (whole TZ extract)	TZ ELISA	E	5	Sows	na	−1 - 12 wpi	CZ clone H3	Oo	10^4	All groups regardless the term of gestation:	[3]
			E	3	Sows	na	−1 - 12 wpi	CZ clone H3	Oo	10^4	IgG seroconversion: 2-3 wpi	
			E	3+2	Sows	na	−1 -12 wpi and −1 - 16 post-insemination	CZ clone H3	Oo	10^4 + $10^5/10^5$	High avidity values: 8 wpi onwards<br>One piglet with low avidity values until 11 wpi	
			E	3	Piglet	na	0-11 wpi	CZ Tiger	Oo			
										$5x10^3$		
	**Assay format (Antigen employed)**	**Reference test**	**Sheep sera**								**Results**	**Ref.**
				**Host-dependent factors**				**Parasite-dependent factors**				
			**(N/E)**	**Nº**	**Age**	**Sheep***	**Samplings**	***T. gondii* strain**	**Stage**	**Dose**		
**IgM tests**	IFAT (TZ)	ELISA/ WB	E	5	8 m	Swedish Landrace	1-5 wpi	M3	Oo	2000	IgM: increased at 2 wpi, peaked at 3 wpi and decreased at 4 and 5 wpi	[4]
**Avidity tests**	ELISA (SAG1)	TZ ELISA	E	6	Ewes	na	0-12 wpi	M3	Oo	2000	High avidity values: 10 wpi onwards	[5]
			N	41	Ewes	na	At slaughter	-	-	-	High avidity values: 90% and 80%, respectively	
			N	and	and	na	At slaughter	-	-	-	High avidity values: 97.4%	

			35	Lambs							
			114	Ewes							
ELISA<br>(crude TZ extract)	TZ ELISA	N	36	na	na	2nd sampling<br>10 m later	-	-	-	High avidity values: 33 animals in both samplings<br>Low avidity values followed by high avidity: 2 sera<br>Low avidity values in both samplings: 1 serum	[6]

Ref: reference; TZ: Tachyzoite; TC: tissue cysts; Oo: Oocysts; N: Natural infection; E: Experimental infection; w: weeks; m: months; SPF: specific pathogen free; wpi: weeks post-infection; dpi: days post-infection; na: no data available; IFAT: Indirect immunofluorescence antibody test; WB: Western blot

** Strain/ breed

* IgG seroconversion is usually detected 2–3 weeks p.i., and detectable levels persist for the duration of the experiments. Kinetics of IgM antibodies have been poorly studied in livestock. They were detected early in pigs but had disappeared by two months p.i. [1], whilst in sheep, specific IgM was found for up to three months p.i. [7]. High IgG avidity indices (indicative of a chronic infection) were seen with a tachyzoite lysate-based ELISA starting 8–10 weeks p.i. in pigs [2,3] and with a SAG1-based ELISA starting 8 weeks p.i. in sheep [5]. However, several open questions remain: (i) do low avidity indices persist for several months, as evidenced in other Sarcocystidae infections; (ii) can IgM levels be detected for several months or years, as it occurs in humans [8], and (iii) can above mentioned kinetics of IgM and IgG avidity responses be extrapolated to the situation of natural infections?

References

1. Lind, P.; Haugegaard, J.; Wingstrand, A.; Henriksen, S.A. The time course of the specific antibody response by various ELISAs in pigs experimentally infected with Toxoplasma gondii. *Vet Parasitol* **1997**, *71*, 1-15, doi:10.1016/s0304-4017(97)00010-1.
2. Suaréz-Aranda, F.; Galisteo, A.J.; Hiramoto, R.M.; Cardoso, R.P.; Meireles, L.R.; Miguel, O.; Andrade, H.F., Jr. The prevalence and avidity of Toxoplasma gondii IgG antibodies in pigs from Brazil and Peru. *Vet Parasitol* **2000**, *91*, 23-32, doi:10.1016/s0304-4017(00)00249-1.
3. Basso, W.; Grimm, F.; Ruetten, M.; Djokic, V.; Blaga, R.; Sidler, X.; Deplazes, P. Experimental Toxoplasma gondii infections in pigs: Humoral immune response, estimation of specific IgG avidity and the challenges of reproducing vertical transmission in sows. *Vet Parasitol* **2017**, *236*, 76-85, doi:10.1016/j.vetpar.2017.01.026.
4. Lundén, A. Immune responses in sheep after immunization with Toxoplasma gondii antigens incorporated into iscoms. *Vet Parasitol* **1995**, *56*, 23-35, doi:10.1016/0304-4017(94)00670-8.
5. Sager, H.; Gloor, M.; Tenter, A.; Maley, S.; Hässig, M.; Gottstein, B. Immunodiagnosis of primary Toxoplasma gondii infection in sheep by the use of a P30 IgG avidity ELISA. *Parasitol Res* **2003**, *91*, 171-174, doi:10.1007/s00436-003-0964-9.
6. Caballero-Ortega, H.; Quiroz-Romero, H.; Olazarán-Jenkins, S.; Correa, D. Frequency of Toxoplasma gondii infection in sheep from a tropical zone of Mexico and temporal analysis of the humoral response changes. *Parasitology* **2008**, *135*, 897-902, doi:10.1017/s0031182008004460.
7. Payne, R.A.; Joynson, D.H.; Wilsmore, A.J. Enzyme-linked immunosorbent assays for the measurement of specific antibodies in experimentally induced ovine toxoplasmosis. *Epidemiol Infect* **1988**, *100*, 205-212, doi:10.1017/s0950268800067339.
8. Ybañez, R.H.D.; Ybañez, A.P.; Nishikawa, Y. Review on the current trends of toxoplasmosis serodiagnosis in humans. *Front Cell Infect Microbiol* **2020**, *10*, 204, doi:10.3389/fcimb.2020.00204.
